# Decreased IgA^+^ B Cells Population and IgA, IgG, IgM Contents of the Cecal Tonsil Induced by Dietary High Fluorine in Broilers

**DOI:** 10.3390/ijerph10051775

**Published:** 2013-05-02

**Authors:** Juan Liu, Hengmin Cui, Xi Peng, Jing Fang, Zhicai Zuo, Junliang Deng, Hesong Wang, Bangyuan Wu, Yuanxin Deng, Kangping Wang

**Affiliations:** Key Laboratory of Animal Diseases and Environmental Hazards of Sichuan Province, College of Veterinary Medicine, Sichuan Agricultural University, Ya’an 625014, China; E-Mails: liujuan9640@163.com (J.L.); pengxi197313@163.com (X.P.); fangjing4109@163.com (J.F.); zzcjl@126.com (Z.Z.); dengjl213@126.com (J.D.); wanghesong0121@163.com (H.W.); wubangyuan2008@163.com (B.W.); dengyuanxin22@163.com (Y.D.); jmq1005@yahoo.com (K.W.)

**Keywords:** fluorine, IgA, IgG, IgM, IgA^+^ B cell, cecal tonsil

## Abstract

Fluoride is an environmental and industrial pollutant that affects various organs in humans and animals. The cecal tonsil is an important component of the mucosal immune system and performs important and unique immune functions. In the present study, we investigated the effects of dietary high fluorine on the quantities of IgA^+^ B cells in the cecal tonsil by immunohistochemistry, and the immunoglobulin A (IgA), immunoglobulin G (IgG) and immunoglobulin M (IgM) contents in the cecal tonsil by ELISA. A total of 280 one-day-old avian broilers were divided into four groups and fed on a corn-soybean basal diet as control diet (fluorine 22.6 mg/kg) or the same diet supplemented with 400, 800 and 1,200 mg/kg fluorine (high fluorine groups I, II and III) in the form of sodium fluoride, respectively, throughout a 42-day experimental period. The results showed that the quantities of IgA^+^ B cells were lower (*p* < 0.05 or *p* < 0.01) and the IgA, IgG, and IgM contents were decreased (*p* < 0.05 or *p* < 0.01) in high fluorine groups II and III in comparison with those of control group. It was concluded that dietary fluorine, in the 800–1,200 mg/kg range, could reduce the numbers of the IgA^+^ B cells and immunoglobulin contents in the cecal tonsil, implying the local mucosal immune function was ultimately impacted in broilers.

## 1. Introduction

Fluoride toxicity is characterized by a variety of signs and symptoms. Generally, poisoning immediately occurs after accidental or intentional ingestion of products which contain fluoride. Fluorosis is a major environmental problem in many regions of the World [1]. Literature reports have shown that ﬂuorosis is endemic in more than 20 developed and developing countries, including Argentina, USA, Morocco, Algeria, Libya, Egypt, Jordan, Turkey, Iran, Iraq, Kenya, Tanzania, S. Africa, China, Australia, New Zealand, Japan, Thailand, Canada, Saudi Arabia, Persian Gulf, Sri Lanka, Syria, India and so on [2]. In China, for example, it is currently estimated that approximately 43 million people have dental ﬂuorosis and 3 million people have skeletal ﬂuorosis [3], due to either high natural background-levels of fluoride [4], or fluoride released from anthropogenic sources [5].

Despite the fact that ﬂuoride has pharmaceutical value, many studies have reported ﬂuoride-induced health disorders. Chronic ﬂuoride toxicity is associated with premature aging, arthritic pain, skeletal and dental ﬂuorosis, osteosclerosis and crippling ﬂuorosis [6]. Acute ﬂuoride toxicity is associated with nausea, salivation and abdominal pain [7]. Moreover, autoimmunity and impairment in cognition and memory are also associated with ﬂuoride intoxication [8]. In addition, fluoride intoxication also results in metabolic disorders [9], renal toxicity [10,11], splenic toxicity [12], thymic toxicity [13] and the variation of blood parameters [14,15].

Fluoride is absorbed in the gastrointestinal tract, so it is well known that the gastrointestinal tract is mainly exposed to fluoride due to the daily food and water consumption. Numerous studies in experimental animals and humans have shown that sodium ﬂuoride (NaF) intake causes gastrointestinal damage [16]. Gastric aberrations are often reported in humans residing in endemic ﬂuoride areas and the symptoms include loss of appetite, nausea, anorexia, ﬂatulence, constipation, and intermittent diarrhea [17]. Studies on ﬂuoride in the gastrointestinal tract mainly focus on its absorption and its injurious effects [18]. As a part of the intestine, the cecal tonsil is located in the proximal of the rectum-cecum-ileum and is the largest lymphoid organ of the avian gut-associated lymphoid tissue, both T and B cells are present in germinal centers [19]. Moreover, the cecal tonsil is an important component of the mucosal immune system and performs important and unique immune function. However, limited data showed effects of fluorine on the immunoglobulin contents in the cecal tonsil in poultry so far. Recently we have reported that dietary high fluorine can induce oxidative damage [20] and decrease the percentages of the T-cell subsets in the cecal tonsil [21].

In the present study, we investigated the local mucosal immune function by detecting the distribution and quantities of IgA^+^ B cells in the cecal tonsil by immunohistochemistry, and changes of immunoglobulin A (IgA), immunoglobulin G (IgG) and immunoglobulin M (IgM) contents in the cecal tonsil by ELISA.

## 2. Materials and Methods

### 2.1. Diets and Broilers

A corn-soybean basal diet formulated by the US National Research Council (NRC) [22] was used as the control diet (fluorine 22.6 mg/kg). Sodium fluoride was mixed into the corn-soybean basal diet to produce three high fluorine diets containing 400, 800, and 1,200 mg/kg fluorine (high fluorine groups I, II, and III), respectively. A total of 280 one-day-old healthy avian broilers were divided into four groups with 70 broilers in each group. Broilers were housed in cages with electrically heated units and were provided with water (fluoride ≤ 1 mg/L) as well as abovementioned diets *ad libitum* for 42 days.

### 2.2. Immunohistochemical Examination for IgA^+^ B Cells in Cecal Tonsil

Five broilers in each group were humanely killed at 14, 28 and 42 days of age for gross examination. After postmortem examination, the cecal tonsil was taken and fixed in 10% neutral buffered formalin, processed and trimmed, and finally embedded in paraffin.

IgA^+^ B cells were localized in the cecal tonsil of broilers by immunohistochemistry. The staining was performed in three different sets to conﬁrm the results. Slices were dewaxed in xylene, rehydrated through a graded series of ethanol, washed in distilled water and phosphate buffer saline (PBS) and then blocked for endogenous peroxidase by incubation with 3% H_2_O_2_ in methanol for 15 m. The sections were subjected to antigen retrieval procedure by microwaving in 0.01 M sodium citrate buffer pH 6.0. Additional washing in PBS was performed before the next 30 min incubation at 37 °C in 10% normal goat serum. The slices were incubated overnight at 4 °C with the diluted (1:100) primary antibodies. The antibodies used were polyclonal mouse anti-chicken IgA heavy chains (SouthernBiotech 8330-01, Birmingham, AL, USA). For negative controls, the slices received PBS in place of the primary antibody. After washing in PBS, the slices were exposed to a 1% biotinylated secondary antibody goat anti-mouse IgG (ZSGB-BIO SP Kit, ZSGB-BIO, Beijing, China) for 1 h at 37 °C. The slices were then incubated with the HRP-streptavidin (ZSGB-BIO SP Kit) for 30 m at 37 °C. To visualize the immunoreaction, sections were immersed in diaminobenzidine hydrochloride (DAB). The reaction was monitored microscopically and stopped by immersion in distilled water, as soon as a brown color staining was visualized. Slices were lightly counterstained with hematoxylin, dehydrated in ethanol, cleared in xylene and mounted.

IgA^+^ B cells were counted using a computer-supported imaging system connected to a light microscope (Olympus AX70, Tokyo, Japan) with a objective magnification of ×40. Then IgA^+^ B cells were quantified by Image-Pro Plus 5.1 (Silver Spring, MD, USA) image analysis software. Each group was measured five slices and each slice was measured five times and averaged. 

### 2.3. Determination of the IgA, IgG and IgM Contents in the Cecal Tonsil by ELISA

At 14, 28, and 42 days of age, five broilers in each group were humanely sacrificed, and the cecal tonsils were immediately removed and chilled to 0 °C in 0.85% NaCl solution. The dissected tonsils were weighed and homogenized in nine volumes of ice-cold 0.85% NaCl solution in a chilled homogenizer, and immediately centrifuged at 3,500 × g at 4 °C. The supernatant fluids were immediately assayed for the IgA, IgG and IgM content by enzyme-linked immunosorbent assay (ELISA) as described by Gaca [23]. The final content was determined by the standard curve and were expressed as μg/mL.

### 2.4. Statistical Analysis

Data of the control and three high fluorine groups were statistically evaluated with SPSS/18.0 software package programme for Windows. Hypothesis testing methods included one way analysis of variance (ANOVA) followed by least significant difference test. *p* < 0.05 was considered as statistical significance. All results were expressed as means ± standard deviation (*X* ± S), representing five broilers in each group. 

## 3. Results

### 3.1. Changes of the IgA^+^ B Cells in the Cecal Tonsil

**Figure 1:**
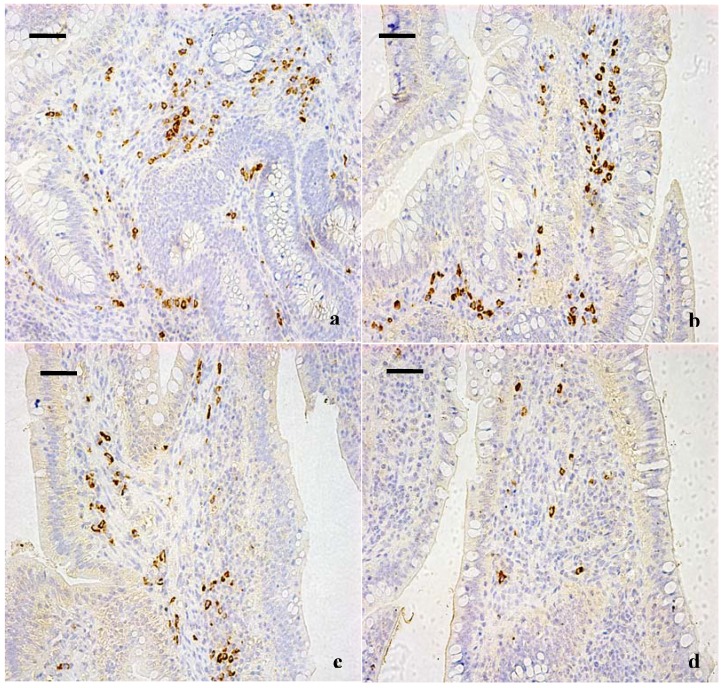
IgA^+^ B cells in the lamina propria of the cecal tonsil at 42 days of age. (**a**) The cecal tonsil in control group (400×, bar = 50 μm); (**b**) The cecal tonsil in high fluorine group I. The quantities of IgA^+^ B cells were not significantly decreased (400×, bar = 50 μm); (**c**) The cecal tonsil in high fluorine group II. The quantities of IgA^+^ B cells were decreased (400×, bar = 50 μm); (**d**) The cecal tonsil in high fluorine group III. The quantities IgA^+^ B cells were significantly decreased (400×, bar = 50 μm).

**Figure 2:**
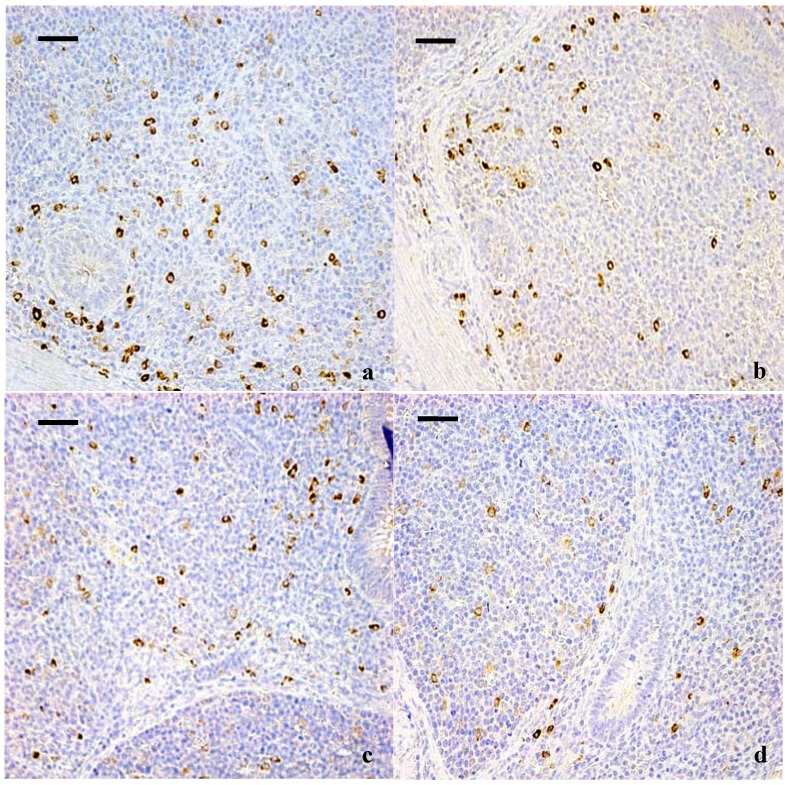
IgA^+^ B cells in the diffuse lymphoid tissues and lymphoid follicles of cecal tonsil at 42 days of age. (**a**) The cecal tonsil in control group (400×, bar = 50 μm); (**b**) The cecal tonsil in high fluorine group I. The quantities of IgA^+^ B cells were not significantly decreased (400×, bar = 50 μm); (**c**) The cecal tonsil in high fluorine group II. The quantities of IgA^+^ B cells were decreased (400×, bar = 50 μm); (**d**) The cecal tonsil in high fluorine group III. The quantities IgA^+^ B cells were significantly decreased (400×, bar = 50 μm).

The results of immunohistochemical determination showed that IgA^+^ B cells mainly distributed in the lamina propria (Figure 1, Figure 2). The positive cells were stained brown. As shown in Table 1, the quantities of IgA^+^ B cells were significantly decreased in high fluorine group I at 42 days and in high fluorine groups II and III at 14, 28 and 42 days of age when compared with those of the control group.

**Table 1:** The number of IgA^+^ cells in cecal tonsil.

Groups	Cecal Tonsil (μg/mL)		
	14 days	28 days	42 days
Control group	190 ± 15	210 ± 18	311 ± 12
High fluorine group I	184 ± 12	189 ± 14	281 ± 14 **
High fluorine group II	97 ± 6 **	147 ± 13 **	134 ± 13 **
High fluorine group III	85 ± 8 **	120 ± 10 **	118 ± 11 **

Data are presented with the means ± standard deviation (n = 5). * *p* < 0.05, compared with the control group. ** *p* < 0.01, compared with the control group.

### 3.2. Changes of the IgA Content in the Cecal Tonsil

A statistically significant difference (*p* < 0.05 or *p* < 0.01) of the IgA content existed in the cecal tonsil between the control group and high fluorine groups II and III during the experimental period. Compared with the control group, this difference was not significant in high fluorine group I (Table 2). 

**Table 2:** Change of the IgA content in broilers.

Groups	Cecal Tonsil (μg/mL)		
	14 days	28 days	42 days
Control group	15.25 ± 0.65	17.03 ± 1.58	19.60 ± 1.00
High fluorine group I	14.23 ± 1.60	17.66 ± 1.41	18.91 ± 1.18
High fluorine group II	13.24 ± 1.25	13.00 ± 1.45 *	12.58 ± 1.38 **
High fluorine group III	12.71 ± 1.50 *	12.13 ± 0.72 **	11.50 ± 1.22 **

Data are presented with the means ± standard deviation (n = 5). * *p* < 0.05, compared with the control group. ** *p* < 0.01, compared with the control group.

**Table 3:** Change of the IgG content in broilers.

Groups	Cecal Tonsil (μg/mL)		
	14 days	28 days	42 days
Control group	7.30 ± 0.49	9.09 ± 0.33	8.70 ± 0.49
High fluorine group I	6.83 ± 0.31	8.83 ± 0.56	8.09 ± 0.51 *
High fluorine group II	6.58 ± 0.60 *	6.18 ± 0.38 **	5.44 ± 0.30 **
High fluorine group III	6.41 ± 0.30 *	5.56 ± 0.10 **	5.22 ± 0.10 **

Data are presented with the means ± standard deviation (n = 5). * *p* < 0.05, compared with the control group. ** *p* < 0.01, compared with the control group.

### 3.3. Changes of the IgG Content in the Cecal Tonsil

Table 3 showed that there was no obvious difference between high fluorine group I and control group. However, the IgG contents in high fluorine groups II and III were decreased (*p* < 0.05 or *p* < 0.01) from 14 to 42 days of age.

### 3.4. Changes of the IgM Content in the Cecal Tonsil

Table 4 clearly showed that the IgM contents were lower (*p* < 0.05) in high fluorine group II at 28 and 42 days of age and in high fluorine group III from 14 to 42 days of age than those in control group.

**Table 4:** Change of the IgM content in broilers.

Groups	Cecal Tonsil (μg/mL)		
	14 days	28 days	42 days
Control group	0.56 ± 0.04	0.52 ± 0.01	0.51 ± 0.01
High fluorine group I	0.54 ± 0.03	0.49 ± 0.01 *	0.50 ± 0.01
High fluorine group II	0.54 ± 0.02	0.49 ± 0.01 *	0.49 ± 0.01 *
High fluorine group III	0.49 ± 0.01 *	0.48 ± 0.02 *	0.48 ± 0.01 *

Data are presented with the means ± standard deviation (n = 5). * *p* < 0.05, compared with the control group. ** *p* < 0.01, compared with the control group.

## 4. Discussion

The mucosal immunity system is a network of gut-associated lymphoid tissue (GALT), bronchial-associated lymphoid tissue (BALT), conjunctiva-assoiated lymphoid tissue (CALT) and genitourinary tract mucous membrane related lymphatic organization (UALT). It is an important and integral component of immune system in the body. It has been reported that fluoride can be an effective adjuvant for mucosal and systemic immunity in rats and chicken, which is closely related to the amount of ﬂuoride. NaF at low concentration may act as an adjuvant but at high concentration may be toxic to animals [5]. Therefore, we investigated the effects of dietary high fluorine on the IgA^+^ B cell population and immunoglobulin contents in the cecal tonsil of broilers in the present study.

The mucosa represents the main portal of entry for viral, bacterial or other pathogens [24]. IgA is the predominant immunoglobulin isotype in the mucosal tissue and is held to be responsible for the defence of mucosal homeostasis [25] and may also influence the development of systemic immunity [26]. Secretory IgA (sIgA) in mucosal secretions can prevent the adsorption of pathogens and neutralize their toxic products at the mucosal epithelium [27], mediate virus neutralization in infected epithelial cells [28], and promote the killing of pathogens through the activation of the alternative pathway of the complement system [29]. The ability of immunoglobulins to protect mucosal surfaces is mainly based on immune exclusion, where binding to pathogens prevents attachment and colonization. Several observations suggest a role for chicken IgA in the protection of mucosal surfaces similar to mammalian IgA [30]. In the present study, the quantities of IgA^+^ B cells and IgA content were significantly reduced in high fluorine groups II and III at 14, 28 and 42 days of age, indicating that high levels of dietary fluorine had inhibition effect on IgA^+^ B cells and sIgA production in the cecal tonsil. 

The B cells that migrate into the lamina propria mature into IgA-producing plasma cells. CD4+ helper T cells act to provide help for B-cell production of IgA while maintaining tolerance to commensal bacteria and possibly other antigens [31]. We have found that dietary high fluorine decreased the population of the CD4+ helper T cells [21], implying that dietary high fluorine can influence the development and maturity of the IgA^+^ B cells due to the reduction of the T cells in the cecal tonsil. IgA-mediated intestinal immune response is controlled by the Th2 type IgA-stimulating and the Th1 type IgA-inhibiting cytokines. Th1 cytokines such as TNF-α and IFN-γ, which enhance cell-mediated immunity, are produced by Th1 lymphocytes. Th2 cytokines such as IL-4 and IL-6, which enhance humoral immunity, are produced by Th2 lymphocytes. The balance between the Th2 and the Th1 type cytokines plays a critical role in IgA control [32]. In our previous study, we found the contents of IL-4 and IL-6 were decreased in the cecal tonsil [33], which may inhibit the production of the IgA^+^ B cells and secretion of the sIgA.

Nowadays, it is assumed that the early maturation phase of the intestinal immune system affects the functionality of the whole immune system later in life [34]. Three classes of chicken immunoglobulins have been identiﬁed immunochemically [35] and genetically [36] as homologues to the mammalian IgM, IgA and IgG. Chicken IgM is structurally and functionally homologous to its mammalian counterpart, being present in serum as a high molecular weight pentamer of μ_2_L_2_ units and being the ﬁrst antibody generated during a primary antibody response. IgM is also the major class of immunoglobulin expressed on the surface of chicken B lymphocytes. In the present study, IgM content was decreased in high fluorine groups II and III at 14, 28 and 42 days of age, which indicated that dietary high fluorine impacted immune defending in the early-stage. IgG from a phylogenetic perspective is equidistant from mammalian IgG and IgE and in birds has sometimes been referred to as IgY. Functionally, IgG is generated mainly in secondary antibody responses and behaves like the chicken homologue of mammalian IgG. In the present study, IgG content was decreased in high fluorine groups II and III, which demonstrated that dietary high fluorine could impact the natural passive immunity. Zhang has reported that NaF could inhibit the production of specific antibodies in human, and the serum IgG and IgM contents were decreased [37]. However, Shen found that serum IgG and IgA content was significantly lower in fluoride exposed workers than those who were not exposed to fluoride, but IgM content was no significant difference [38]. The difference of these results may be related to the subjects of individual differences, environment conditon, exposure time, and many other factors. NaF inhibited the antibody formation by inhibiting the DNA and protein synthetic ability of immunocytes and by decreasing the proliferation of lymphocytes. The increasing of lymphocyte apoptosis rate decreased the number of plasma cells which were transformed by B lymphocytes, finally affected the number of immunoglobulin content [39]. In our previous study, we have found that dietary fluorine in the range of 800–1,200 mg/kg increased lymphocyte apoptosis in cecal tonsil, and mediated by direct effects of fluoride on the expression of bcl-2, bax and caspase-3 [40].

## 5. Conclusions

According to the results obtained in the present study and the aforementioned discussion, it is concluded that dietary fluorine, in the range of 800–1,200 mg/kg, reduces the quantities of IgA^+^ B cells and the IgA, IgG and IgM contents in the cecal tonsil, implying the local mucosal immune function can be finally impacted in broilers.

## References

[1] Kanbur M., Eraslan G., Silici S., Karabacak M. (2009). Effects of sodium fluoride exposure on some biochemical parameters in mice: Evaluation of the ameliorative effect of royal jelly applications on these parameters. Food Chem. Toxicol..

[2] Mameri N., Yeddou A.R., Lounici H., Belhocine D., Grib H., Bariou B. (1998). Defluoridation of septentrional Sahara water of North Africa by electrocoagulation process using bipolar aluminium electrodes. Water Res..

[3] Ba Y., Huang H., Yang Y.J., Cui L.X., Zhu J.Y., Zhu C.R., Liu J., Zhang Y.W. (2009). The association between osteocalcin gene polymorphism and dental fluorosis among children exposed to fluoride in People’s Republic of China. Ecotoxicol. Environ. Saf..

[4] Garrott R.A., Eberhardt L.L., Otton J.K., White P.J., Chaffee M.A. (2002). A geochemical trophic cascade in Yellow stone’s geothermal environments. Ecosystems.

[5] Sosroseno W. (2003). Effect of sodium fluoride on the murine splenic immune response to porphyromonas gingivalis *in vitro*. Immunopharmacol. Immunotoxicol..

[6] Pak C.V.C., Sakhre K., Zorwekh J.E., Parcel C., Peterson R., Johnson K. (1989). Safe and effective treatment of osteoporosis with intermittent slow release of sodium fluoride: Augmentation of vertebral bone mass and inhibition of fractures. J. Clin. Endocrinol. Metab..

[7] Whitford G.M. (1992). Acute and chronic fluoride toxicity. J. Dent. Res..

[8] Gibson S. (1992). Effect of fluoride on immune system function. Complement. Med. Res..

[9] Liu X.L., Li C.C., Liu K.J., Cui C.Y., Zhang Y.Z., Liu Y. (2012). The influence of fluoride on the expression of inhibitors of Wnt/β-Catenin signaling pathway in rat skin fibroblast cells. Biol. Trace Elem. Res..

[10] Bai C.M., Chen T., Cui Y., Gong T., Peng X., Cui H.M. (2010). Effect of high fluoride on the cell cycle and apoptosis of renal cells in chickens. Biol. Trace Elem. Res..

[11] Nabavi S.F., Moghaddam A.H., Eslamim S., Nabavi S.M. (2012). Protective effects of curcumin against sodium fluoride-induced toxicity in rat kidneys. Biol. Trace Elem. Res..

[12] Chen T., Cui H.M., Cui Y., Bai C.M., Gong T. (2010). Decreased antioxidase activities and oxidative stress in the spleen of chickens fed on high-fluorine diets. Hum. Exp. Toxicol..

[13] Chen T., Cui H.M., Cui Y., Bai C.M., Gong T. (2010). Cell-cycle blockage associated with increased apoptotic cells in the thymus of chickens fed on diets high in fluorine. Hum. Exp. Toxicol..

[14] Chen T., Cui Y., Bai C.M., Gong T., Peng X., Cui H.M. (2009). Decreased percentages of the peripheral blood T-cell subsets and the serum IL-2 contents in chickens fed on diets excess in fluorine. Biol. Trace Elem. Res..

[15] Bharti V.K., Srivastava R.S. (2012). Effect of pineal proteins at different dose level on fluoride-induced changes in plasma biochemical and blood antioxidants enzymes in rats. Biol. Trace Elem. Res..

[16] Blaszczyk L., Birkner E., Gutowska I., Romuk E., Chlubek D. (2012). Influence of methionine and vitamin E on fluoride concentration in bones and teeth of rats exposed to sodium fluoride in drinking water. Biol. Trace Elem. Res..

[17] Susheela A.K., Kharp P. (1990). Aortic calcification in chronic fluoride poisoning: Biochemical electron microscopic evidence. Exp. Mol. Pathol..

[18] Gharzouli K., Amira S., Khennouf S., Gharzouli A. (2010). Effects of sodium fluoride on water and acid secretion, soluble mucus and adherent mucus of the rat stomach. Can. J. Gastroenterol..

[19] Lillehoj H.S., Trout J.M. (1996). Avian gut-associated lymphoid tissues and intestinal immune responses to Eimeria parasites. Clin. Microbiol. Rev..

[20] Liu J., Cui H.M., Peng X., Fang J., Zuo Z.C., Wang H.S., Wu B.Y., Deng Y.X., Wang K.P. (2012). Dietary high fluorine induces oxidative damage in the cecal tonsil of broilers. Fluoride.

[21] Liu J., Cui H.M., Peng X., Fang J., Zuo Z.C., Wang H.S., Wu B.Y., Deng Y.X., Wang K.P. (2012). Decreased percentages of T-cell subsets and IL-2 contents in the cecal tonsil of broilers fed diets high in fluorine. Fluoride.

[22] (1994). Subcommittee on Poultry Nutrition, Committee on Animal Nutrition, Board on Agriculture, National Research Council—Ninth Revised Edition.

[23] Gaca M.D.A., Pickering J.A., Arthur M.J.P., Benyon R.C. (1999). Human and rat hepatic stellate cells produce stem cell factor: A possible mechanism for mast cell recruitment in liver fibrosis. J. Hepatol..

[24] Kaul D., Ogra P.L. (1998). Mucosal responses to parenteral and mucosal vaccines. Dev. Biol. Stand..

[25] Cerutti A., Rescigno M. (2008). The biology of intestinal immunoglobulin A responses. Immunity.

[26] Favre L., Spertini F., Corthesy B. (2005). Secretory IgA possesses intrinsic modulatory properties stimulating mucosal and systemic immune responses. J. Immunol..

[27] Mazanec M.B., Nedrud J.G., Kaetzel C.S., Lamm M.E. (1993). A three-tiered view of the role of IgA in mucosal defense. Immunol. Today.

[28] Mazanec M.B., Kaetzel C.S., Lamm M.E., Fletcher D., Nedrud J.G. (1992). Intracellular neutralization of virus by immunoglobulin A antibodies. Proc. Natl. Acad. Sci. USA.

[29] Janoff E.N., Fasching C., Orenstein J.M., Rubins J.B., Opstad N.L., Dalmasso A.P. (1999). Killing of Streptococcus pneumoniae by capsular polysaccharide-specific polymeric IgA, complement, and phagocytes. J. Clin. Invest..

[30] Wieland W.H., Orzaez D., Lammers A., Parmentier H.K., Verstegen M.W.A., Schots A. (2004). A functional polymeric immunoglobulin receptor in chicken (*Gallus gallus*) indicates ancient role of secretory IgA in mucosal immunity. Biochem. J..

[31] Mowat A.M. (2003). Anatomical basis of tolerance and immunity to intestinal antigens. Nature Rev. Immunol..

[32] Kudsk K.A. (2001). Importance of enteral feeding in maintaining gut integrity. Tech. Gastrointest. Endosc..

[33] Liu J., Cui H.M., Peng X., Fang J., Zuo Z.C., Wang H.S., Wu B.Y., Deng Y.X., Wang K.P. (2012). Changes induced by high dietary fluorine in the cecal tonsil cytokine content of broilers. Fluoride.

[34] Noble A. (2009). Do we have memory of danger as well as antigen?. Trends Immunol..

[35] Leslie G.A., Martin L.N. (1973). Studies on the secretory immunologic system of fowl. III. Serum and secretory IgA of the chicken. J. Immunol..

[36] Mansikka A. (1992). Chicken IgA H chains. Implications concerning the evolution of H chain genes. J. Immunol..

[37] Zhang A.J., Yang D.M., Bai S.Q. (2002). The immune toxicity of fluoride. Chin. J. Ctrl. End..

[38] Shen L.X., Liu J.D., Chen R.A. (1996). The changes of serum IgA, IgG, and IgM content in fluoride exposed worker. Chin. J. Ind. Med..

[39] Jain S.K., Susheela A.K. (1987). Effect of sodium fluoride on antibody formation in rabbits. Environ. Res..

[40] Liu J., Cui H.M., Peng X., Fang J., Zuo Z.C., Wang H.S., Wu B.Y., Deng Y.X., Wang K.P. (2013). Dietary high fluorine induces apoptosis and alters bcl-2,bax, and caspase-3 protein expression in the cecal tonsil lymphocytes of broilers. Biol. Trace Elem. Res..

